# A Methodology to Evaluate Long Term Durability of Dam Concrete Due to Calcium Leaching through Microscopic Tests and Numerical Analysis

**DOI:** 10.3390/ma14247819

**Published:** 2021-12-17

**Authors:** Ding Nie, Haoyu Wang, Pengfei Li, Xun Han, Jingbin Zhang, Chengzhi Wang

**Affiliations:** 1 State Key Laboratory of Simulation and Regulation of Water Cycle in River Basin, China Institute of Water Resources and Hydropower Research, Beijing 100038, China; nieding@iwhr.com; 2 Department of Harbor, Waterway, and Coastal Engineering, Chongqing Jiaotong University, Chongqing 400074, China; why0422@mails.cqjtu.edu.cn (H.W.); wangcz@cqjtu.edu.cn (C.W.); 3 Geotechnical Engineering Department, Nanjing Hydraulic Research Institute, Nanjing 210029, China; xhan@nhri.cn; 4 College of Civil and Transportation Engineering, Hohai University, Nanjing 210098, China; zhangjingbin_hhu@163.com

**Keywords:** concrete dams, calcium leaching, multi-scale numerical models, numerical analysis, microscopic tests

## Abstract

Hydropower dams are subjected to soft water penetration during their service lives. Concrete deterioration due to calcium leaching will decrease the durability of concrete and affect dam safety. The long-term performance of concrete dams due to calcium leaching should be evaluated and predicted accurately to complete reinforcement work in a timely manner. In this paper, a methodology that combined microscopic tests and numerical analysis to evaluate the long-term performance of dam concrete due to calcium leaching is proposed. The current state of concrete is evaluated by analyzing the components of sediments and seepage water through microscopic and spectroscopic tests, such as X-ray photoelectron spectroscopy, scanning electron microscopy, and inductively coupled plasma mass spectrometry. The long-term degradation of concrete was predicted by utilizing a multi-scale model of calcium leaching, which considered the micro-pore structure of cement hydrates flux with time. The simulated results using this calcium leaching model showed a good agreement with other experiments. Finally, a real case study including field inspection was performed and the long-term durability of dam concrete was predicted through microscopic tests and finite element analysis method. It implies that the proposed method could provide calculation and theoretical basis for the durability analysis of concrete dams due to calcium leaching.

## 1. Introduction

Concrete dams are designed to store water, mostly soft water, throughout their lifetime. Calcium leaching of dam concrete is common in China because reservoir water is usually soft and has medium soluble corrosivity [1]. At the macro-level, these concrete structures are prone to problems, such as loss of strength, performance degradation, and shortened service life under long-term soft water penetration [2,3]. At the micro-level, this phenomenon is due to calcium ions in the concrete pore solution diffusing into the soft water under the concentration gradient, causing a soluble phase of cement hydration products, such as calcium hydroxide (Ca(OH)_2_), calcium-silicate-hydrate gel (C-S-H gel) and other solid calcium, to dissolve and be lost [4,5]. Under normal leaching conditions, calcium leaching of concrete is extremely slow due to the diffusion-controlled process [6]. However, when decreasing pH [7], increasing in transport properties [8], increasing in porosity [9,10], and other properties changing [6] in concrete due to mechanical or other effects, such as undesigned cracks, calcium leaching rate might be significantly altered. As time passes, the calcium concentration and alkalinity of the pore solution inside the concrete decrease, while porosity increases, causing a loss of strength and impermeability in concrete [11,12,13]. For concrete dams, according to the Chinese national standard [14], the design lifetime often exceeds 100 years or more. Calcium leaching over such a long service life becomes an essential issue to be considered in the design, operation, and maintenance period of such projects. Abandonment of the Colorado arch dam, built in 1912 in the USA, was mainly due to calcium leaching according to field inspections. Investigations of 34 dams, including masonry gravity dams, masonry arch dams, concrete arch dams, and concrete gravity dams, also show that calcium leaching is one of the main deterioration factors of dams in China [1]. Therefore, it is of great importance to estimate and predict the properties of concrete at a later stage, after construction, to promptly complete the reinforcement work and ensure a dam’s long-term safety.

In order to investigate the current state of leaching of concrete, field inspections, including visual inspection, sampling of leaching deposits together with seepage water and samples analyses, must be done. The combined utilization of microscopic experimental methods, such as X-ray diffraction (XRD), X-ray photoelectron spectroscopy (XPS), scanning electron microscopy (SEM), and inductively coupled plasma mass spectrometry (ICP-MS), is an effective and quantitative method to analyze deposits and water samples. The chemical elements and mineral components of leaching precipitates and the dosages of calcium and other ions in seepage water could be detected quantitatively using these microscopic techniques. Rosenqvist et al. [15] analyzed chemical and mineralogical modifications of cement paste from a concrete buttress dam after 55 years of exposure to water using XRD, SEM, and energy dispersive X-ray spectrometry (XRS) methods. Pathak et al. [3] used XRD and other microscopic tests to characterize leachate materials from a concrete dam. Karandashev et al. [16] analyzed calcium and other ions from natural and potable water using ICP-MS. All these microscopic tests provided effective and reliable tools to quantitatively detect the components of leaching precipitates and seepage water. Thus, the current leaching state of dam concrete could be evaluated based on test data.

Recently, many researchers have investigated experimental studies on the calcium dissolution process of hardened cementitious materials to propose numerical calcium leaching models. These studies have used accelerated experimental methods, including the application of an electrical field [17], use of deionized water [18], or low-pH solutions [19] to shorten the experiment time. However, according to the inspection results from real dams, the calcium leaching rate of cementitious materials under soft water is quite a slow process. Thus, prediction and analysis of this phenomenon are difficult when only using normal or accelerated experiments. Experimental methods need to be taken into consideration that combine numerical methods for a more accurate evaluation of concrete properties since real-time scale tests are almost unrealistic. Many researchers have proposed numerical calcium leaching models to calculate the degradation of cement-based materials. Most models focus on the prediction of the leached depth of leached materials [20] and characterize their mechanical behavior [21]. Samon et al. [22] presented a multi-ionic transport model for saturated and unsaturated cementitious materials. Li et al. [23] developed a one-dimensional model of calcium ion transportation. Perko et al. [4] proposed a coupled numerical model to evaluate the influence of pore connectivity and microcracks on leaching kinetics in fully saturated cement paste. All these models provide a theoretical basis and numerical methods to analyze and simulate a long-term calcium leaching process. Considering the micropore structures are assumed to be constant through all leaching processes in these models, while calcium leaching and the evolving microstructure are strongly coupled with each other, both need to be simulated, especially over extremely long periods of time. Hence, a multi-scale model, proposed by Nakarai et al. [24], integrating a calcium ion transport model with a statistical micro-structural model of chemophysics, was used in this study.

In this paper, a methodology that combines microscopic tests and numerical analyses is proposed to evaluate the long-term durability of dam concrete due to calcium leaching. Firsty, field inspections, together with the utilization of specific microscopic experimental tests, could provide a quantitative method to evaluate the current degrees of calcium leaching. The experimental results also provided boundary and material parameters for numerical analyses. Then, the calcium leaching model and its numerical system were comprehensively introduced, providing numerical ways to analyze and predict the long-term durability of concrete. Verifications of the concrete immersion tests proved that this model shows good agreement with the experiments. Finally, a real case study, including field inspection of a concrete gravity dam, was conducted. Concrete dissolution was found in the dam by detecting the components of sediments and seepage water collected on-site. According to the results of the test, long-term durability prediction of the dam concrete could be performed and reasonable advice was drawn based on the numerical analysis results.

## 2. Methodology

### 2.1. Total Framework

**Figure 1:**
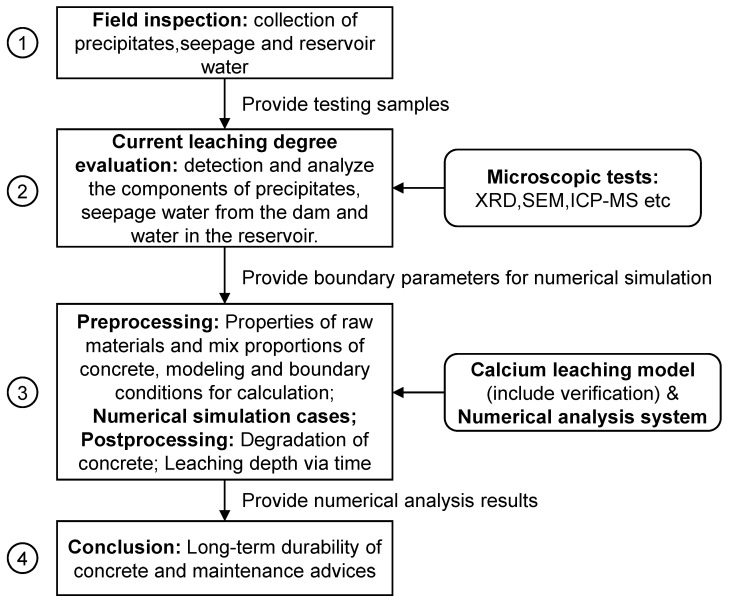
The total framework of methodology to evaluate the long-term durability of dam concrete due to calcium leaching.

Figure 1 illustrates the total framework of the methodology to evaluate the long-term durability of dam concrete due to calcium leaching. The basic steps included field inspections, current leaching degree evaluation, numerical analyses, and conclusions.

At first, a field inspection must be conducted to check the leaching degree and collect samples on-site. Leached calcium ions (Ca^2+^) from the concrete inside the dam were dissolved in water that penetrated into the dam from the reservoir. The penetration process is mostly associated with construction joints or cracks. The formation of leached Ca^2+^ forms Ca(OH)_2_, which ultimately forms calcium carbonate (CaCO_3_) on the surface of the concrete through the reaction of carbon dioxide (CO_2_) in the atmosphere, as shown in Section 3.3. The minerals components and chemical elements of the leaching materials, including solid deposits and seepage water, directly indicate the leaching degree of dam concrete. In addition, leaching in dam concrete may carry other minerals, such as quartz, aluminates, etc., from the core material, apart from the CaCO_3_. Hence, in order to evaluate the current degree of Ca^2+^ leaching, both the solid precipitates and seepage water inside the dam, together with the water in the reservoir, should be collected on-site for ingredient detection.

Through comprehensive analyses of the leaching precipitates, together with the ions’ viability in the seepage water and reservoir water, the current leaching degree of dam concrete could be assessed. Though the current degree of calcium leaching was slight, the long-term durability of concrete could still be serious, considering the mixed proportions of dam concrete, pH value, and aggressive ions in the reservoir water. Thus, the long-term performance of dam concrete could be predicted based on the calcium leaching model and numerical analysis system used in this study. Considering the investigation results from microscopic tests and design blueprints of the hydropower station, the properties of raw materials, mix proportions of concrete, simplified numerical model, and boundary conditions for the calculations could be confirmed.

After finishing several cases of numerical simulations, the degradation of dam concrete and leaching depth versus time are processed. Finally, the durability of concrete versus time and maintenance advice can be drawn based on the entire process.

### 2.2. Field Inspection, Precipitates, and Water Sample Analysis

Field inspections, including visual inspections, mainly allowed engineers to verify dam seepage status, calcium leaching, or other corrosion at the site. During field inspections, solid deposits and seepage water were sampled from the downstream surface, corridor walls, and drain pipes. Reservoir water was collected from different depths in the reservoir near the upstream surface of the dam. Deposits, seepage water, and reservoir water were sampled, sealed, and marked in the field. All the samples needed to be analyzed in a laboratory to allow a comprehensive evaluation of the dam concrete’s current status.

Chemical and mineralogical modifications of the deposit samples were analyzed using XRS, XPS, and infrared spectrophotometry (IS) combined with SEM in a laboratory. Mineralogical identification of the deposits was done using XRD and IS. Chemical constituents were analyzed and determined using XPS. SEM images allowed to identify the microstructure of the leached deposits for auxiliary verification of chemical and mineralogical analysis results. Current studies show that corrosion types of water on concrete can be classified into three categories: soluble corrosion, acid corrosion, and carbonic acid corrosion. Corrosion types could be classified through the chemical elements and pH value of water. Hence, chemical analyses of both reservoir and seepage water needed to be conducted, following standard test methods. The corrosion types could be judged through the compositions and pH values of the water. In this study, soluble corrosion due to soft water was the main effect according to the water sample analysis results. The soluble corrosion or calcium leaching rate should be predicted using numerical methods based on field inspections and laboratory analysis results.

### 2.3. Test Equipment and Parameters

First, the composition of the concrete deposits of the dam was analyzed with an X-ray diffractometer, type D8ADVANCE, manufactured by Bruker in Massachusetts, USA. The diffraction peak data were obtained, including peak height, position, crystal surface spacing, and relative intensity of the deposits. Jade software was used to determine the composition of the deposits by comparing the peaks of the XRD spectrum to those of the standard pattern.

Next, to study the micromorphology of sediments, SEM experiments using a JSM-7401F field emission scanning electron microscope manufactured by JEOL Ltd. in Tokyo, Japan were used. The deposit samples were prepared by soaking in ethanol for 24 h and then drying at 105 °C for 48 h.

Finally, in order to study the chemical elemental species and content of the sediments, XPS was carried out using a 250XI X-ray photoelectron spectrometer from Thermo Scientific in Massachusetts, USA, which had an optimum energy resolution less than or equal to 0.45 eV and a sensitivity of greater than or equal to 400,000 cps. A standard sample of the solid sediment was prepared and placed in the test instrument for detection.

### 2.4. Numerical Simulation Process

#### 2.4.1. Numerical Analytical System

In accordance with previous researchers, Ca^2+^ leaching from cement hydrates has a strong relationship with the process of microstructure formation, cement hydration, and moisture distribution. All these chemo-physical processes are highly time dependent. According to a theoretical study, a computational method was developed by Maekawa et al. [25,26,27] for numerical simulations, and the computer program is called DuCOM (durability of the concrete model). Based on physical chemistry, DuCOM can predict thermodynamic states and micro-pore structures in multi-scale pores, considering hydration and environmental actions. This method is used in this study to verify the Ca^2+^ leaching model and to analyze the long-term properties of dam concrete. The DuCOM numerical analysis system consists of a multi-component hydration model, a moisture transport/equilibrium model, and a micro-pore structure development model. In addition to the above-mentioned computational models, the calcium leaching model was the basis for this study. It relates the total mass of calcium in pore solution and the solid phase calcium in the system. This system enables the strong interaction between calcium leaching and the micro–macro solid features of concrete to be consistently taken into account.

#### 2.4.2. Modeling of Calcium Leaching

Based on the method of DuCOM, Nakarai [24] developed a new model for Ca^2+^ leaching, coupled with time-dependent material properties. This model follows the law of mass conservation and combines the equilibrium of calcium in the solid and liquid phases with the Ca^2+^ transport of both diffusion and advection.

As the governing equation for calcium, the mass conservation equation was applied in terms of the total calcium ions in the pore solution and the solid-phase calcium. The calcium leaching model of Ca^2+^ in pore solution is presented in Equation (1), as per Gerard et al. [28].

(1) $$\frac{\partial }{\partial t}\left(\phi \cdot S\cdot C_{ion}\right)+\frac{\partial C_{solid}}{\partial t}-divJ_{ion}=0,$$

where *ϕ* is porosity, *S* is the degree of saturation, *C_ion_* is the concentration of Ca^2+^ in the liquid phase, *C_solid_* is the amount of calcium in the solid phase, and *J_ion_* is the flux of Ca^2+^. The porosity and the saturation are calculated in the DuCOM system. The next step is to try to calculate the solid calcium and the liquid Ca^2+^.

The mathematical formulation adopted by Gerard et al. [28] and later modified was referred to as,

(2) $$C_{solid}=f\left(C_{ion}\right)=AC_{CSH}{\left(\frac{C_{ion}}{C_{satu}}\right)}^{1/3}+B,$$

(3) $$A=\left\{ \begin{array}{ll} -\frac{2}{x_{1}^{3}}C_{ion}^{3}+\frac{3}{x_{1}^{2}}C_{ion}^{2} & \left(0\leq C_{ion}\leq x_{1}\right)\\ 1 & \left(x_{1}\leq C_{ion}\right) \end{array}\right.,B=\left\{ \begin{array}{ll} 0\; & \left(0\leq C_{ion}\leq x_{2}\right)\\ \frac{C_{CH}}{{\left(C_{satu}-x_{2}\right)}^{3}}\;{\left(C_{ion}-x_{2}\right)}^{3} & \left(x_{2}\leq C_{ion}\right) \end{array}\right.,$$

where *C_CSH_* is the amount of calcium in the solid phase of the C-S-H gel, *C_CH_* is the amount of calcium in the solid phase of the calcium hydroxide, *C_satu_* is the saturated liquid phase Ca^2+^ concentration. According to Nakarai’s research, *x*_1_ and *x*_2_ are 0.3 mmol/L and (*C_satu_* − 0.7) mmol/L, respectively. All the parameters are calculated as time-dependent variables under the scheme of DuCOM.

Nakarai’s study showed that the flux of Ca^2+^ transported in a porous media is written as,

(4) $$J_{ion}=-\left(\frac{\phi \cdot S}{\tau }\cdot \delta \cdot D_{ion}\right)\cdot \nabla C_{ion}+\phi \cdot S\cdot u\cdot C_{ion},$$

where *τ* is tortuosity, *δ* is constrictivity, *D_ion_* is the diffusion coefficient of a Ca^2+^, and **u** is the velocity vector of Ca^2+^ transported by the solution flow. The calculation of the tortuosity factor and constructive factor can be found in Maekawa’s study [27].

#### 2.4.3. Verification of the Calcium Leaching Model

In order to verify the calcium leaching model, in addition to the verification from their study, another verification by simulating cement paste immersion tests, conducted by Samson et al. [22], is provided in this paper.

In Samson’s experiment, cement pastes were prepared at a water/cement ratio of 0.6 by mass and then cast into cylinders with a diameter of 7 cm and a height of 20 cm. After the hydration period, the cylinders were sawed in disks with a height of 2 cm. Before being immersed in water, the disks were sealed on the sides and on one face with silicon. All the test disks were immersed in deionized water for at least 3 months. After the pre-processing procedure, the degradation states of the test samples, including the total calcium contents, were analyzed using a microprobe.

The input parameters, including mixture proportions and mineral compositions of cement, are listed in Table 1. Among them, C_3_A represents tricalcium aluminate, C_3_S represents tricalcium silicate, C_2_S represents dicalcium silicate, and C_4_AF represents tetra calcium iron aluminate. A one-dimensional FE model was established to simulate the calcium ion (dissolved) migration and leaching from Ca(OH)_2_ and C-S-H gel. The boundary conditions were set to be the same as the experiment. The exposed face of the disk (at *x* = 0) was set to transfer heat, humidity, and calcium ions, and all concentrations were set to 0 either. The rest faces were set to transfer only heat. Time steps of 30 min were used and the total calculation time was 90 days.

**Figure 2:**
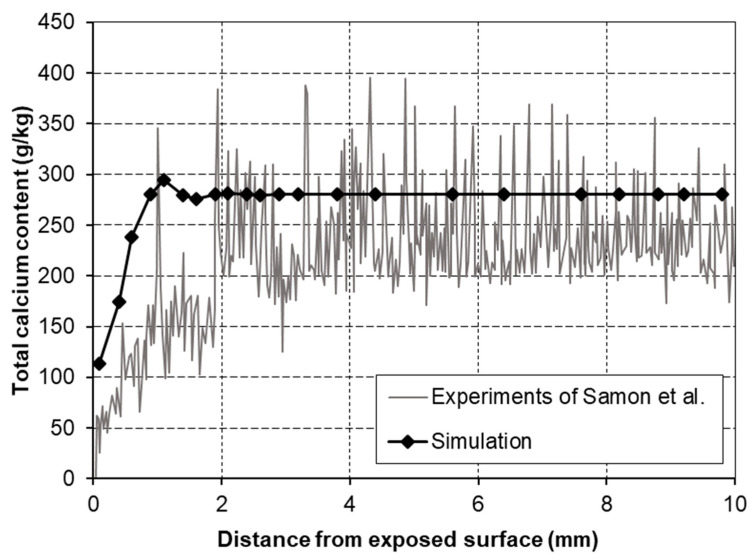
The experimental and simulated profiles of calcium distribution.

In Figure 2, the numerical simulation profiles of total calcium content are plotted, together with the experimental profiles. The simulation demonstrates slightly less deterioration than that found from the experiments. The sealed side surfaces may have had a slight leaching of calcium ions, accelerating deterioration in the experiment. This little discrepancy was considered to be acceptable. For a more accurate estimate, it would be necessary to modify the boundary conditions and improve the parameters in the model. According to these comparisons, it can be concluded that the former verifications all showed good applicability of the transport model in DuCOM for calculating and analyzing calcium leaching from cementitious materials.

## 3. Results

In this part, sediments and seepage from a concrete gravity dam were detected and analyzed. The DuCOM system and the calcium leaching model were applied to carry out numerical analyses and predictions of the long-term properties of the dam concrete.

### 3.1. Field Exploration and Sampling

**Figure 3:**
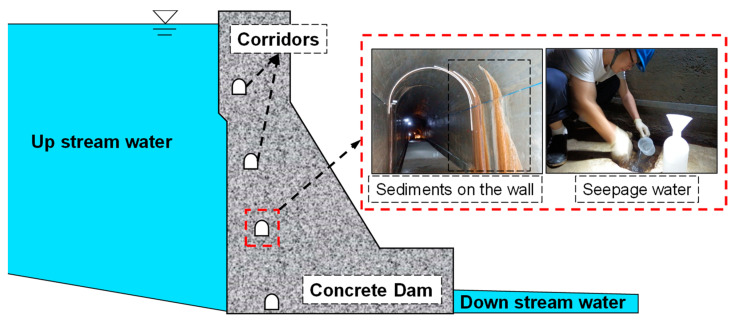
The experimental and simulated profiles of calcium distribution.

The concrete dam of the hydropower station is located in Southeastern China. After 10 years of operation, precipitates, including sediments and seepage water, were found in the corridors inside the dam, as shown in Figure 3. In order to evaluate the long-term deterioration of the dam’s concrete, the precipitates inside the dam needed to be detected and analyzed first.

Both the upstream and corridor seepage water were collected and analyzed according to Chinese national standards. The dosages of calcium and pH value were detected quantitatively using inductively coupled plasma mass spectrometry and the glass electrode method, respectively. The average calcium ion dosages and pH values of upstream water and corridor seepage water were 1.45 mg/L and 21.37 mg/L, respectively. The relevant average pH values were 6.58 and 10.42, respectively. Increases in calcium ions and pH value were observed from the upstream and the corridor.

### 3.2. Detection and Analysis of Seepage Water

In front of dam sections #5, #7, and #9, selecting three vertical lines on the cross-section of the reservoir of about 50 m above the dam surface, a total of nine samples were collected at 0.2–0.5 m below the surface of the reservoir and in the middle of the water depth, and near the bottom of the reservoir, respectively. Two samples were taken from the drainage holes in the foundation of dam section #7. The test results are shown in Table 2.

According to the test results, the calcium content and pH value upstream of the dam and in the corridor showed an upward trend, from which it can be inferred that calcium leaching occurred to a certain extent during the seepage process of the reservoir water through the concrete inside the dam.

### 3.3. Detection and Analysis of Sediments

**Figure 4:**
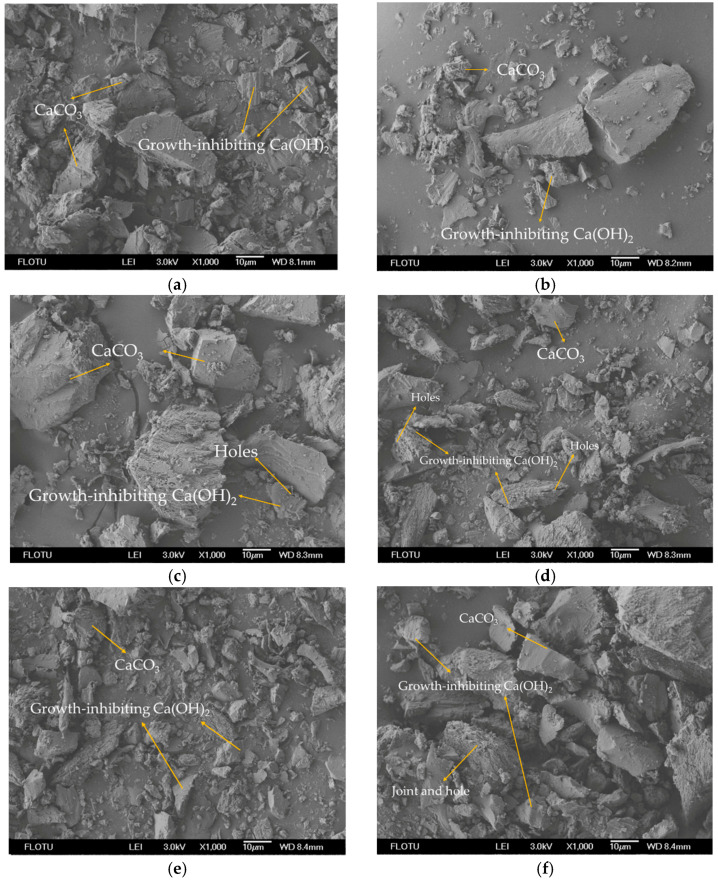
SEM morphology image (×1000) of solid sediments: (**a**) #1 sediment; (**b**) #2 sediment; (**c**) #3 sediment; (**d**) #4 sediment: (**e**) #5 sediment; (**f**) #6 sediment.

A total of six samples were collected from the leakage point of the #7 dam section corridor and from solid sediments in the corridor pipeline, respectively. XPS and SEM were used to detect the composition of solid sediments collected from the corridor. The elemental composition and micromorphology of the samples are shown in Figure 4.

Among them, samples #1–#3 were taken from the leakage point of the #7 dam section corridor, and samples #4~#6 were taken from the solid sediments in the corridor pipeline (removed). As can be seen in Figure 4, the precipitates were mainly irregular prisms, with a size range of approximately 10–20 μm. The crystal surface was porous, inhibiting growth. In Figure 4, each sample contained a large number of crystals with inhibited growth, while a small number of calcium hydroxide crystals can be seen in Figure 4a–c.

**Figure 5:**
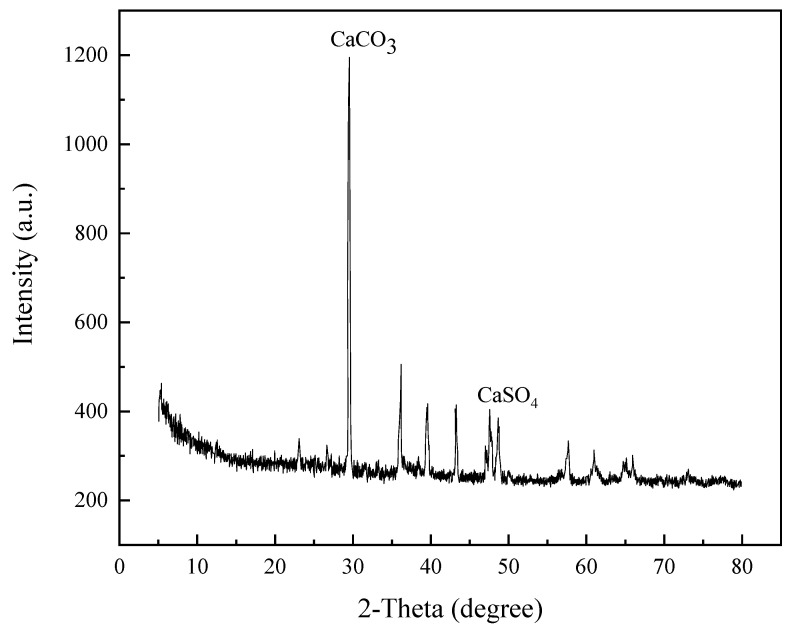
Mineralogical characterization result of representative solid sediments.

In fact, the product of the dissolution reaction is mainly calcium hydroxide, but the presence of a small number of sulfate ions (SO_4_^2−^) in the pore solution, inside the concrete, will change the behavior of the calcium hydroxide. In the XRD analyses in Figure 5, it can be seen that one of the chemical compositions of the precipitate was CaSO_4_. Studies [29,30] have shown that the crystal form of calcium hydroxide at normal temperature is hexagonal, and its particle size ranges from 10 to 20 μm. The presence of SO_4_^2−^ will inhibit the growth of calcium hydroxide crystals, which results in a large number of small plate-like particles and pores on the surface of particles [31].

It can be seen that the main component of the precipitate was calcium hydroxide. Considering that the reservoir water is weakly acidic, part of the calcium hydroxide may have been converted into calcium carbonate precipitate. Furthermore, it can be seen from Figure 5 that the main chemical component of the precipitates was CaCO_3_. This view was confirmed by XRD analysis.

**Figure 6:**
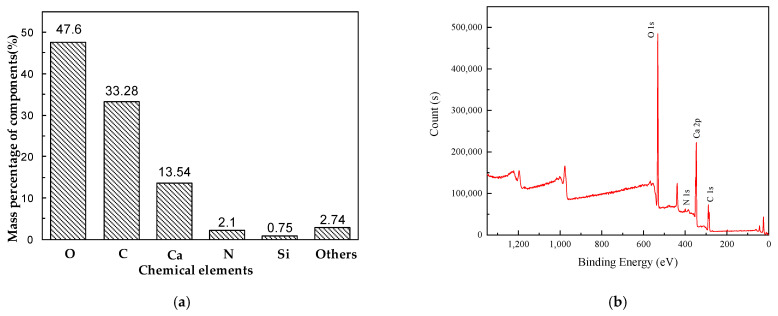
XPS analysis results: (**a**) the mass percentage of main components and (**b**) a representative XPS spectra graph.

In order to further determine the composition of he precipitates, XPS analysis was carried out for the above-mentioned sediments, and the results are shown in Figure 6. As shown in Figure 6a, the main element mass fractions, from high to low, are oxygen (O), carbon (C), calcium (Ca), nitrogen (N), and the remaining elements were less than 1%. Figure 6 shows the average mass percentage of elements coming from six independent measurements. The standard deviations for O, C, Ca, and N were 1.98, 2.07, 1.11, and 0.38 respectively. A representative XPS spectrum is shown in Figure 6b, from which the presence of O 1s, N 1s, Ca 2p, and C 1s peaks in the sediment sample was evident. This is the same as above based on the SEM analysis results.

Combined with the micromorphologies and the elements mass percentage of the components, solid precipitates might consist of calcium carbonate and grouting materials. The calcium carbonate was probably formed by calcium hydroxide caused by calcium leaching dissolution. Based on the repair record, the grouting material was polyurethane, which is mainly composed of carbon and oxygen. The detection and analysis of liquid and solid precipitates all indicated that a certain degree of calcium leaching dissolution existed in the concrete inside the dam.

### 3.4. Numerical Analysis of Dam Concrete by DuCOM

**Figure 7:**
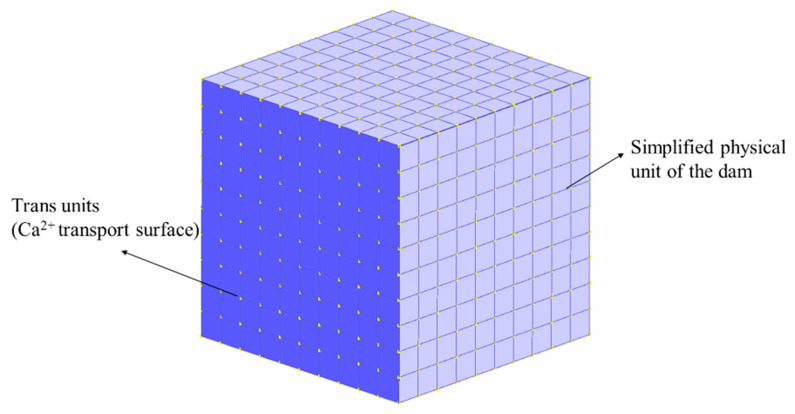
Simplified DuCOM calculation model for dam concrete.

In this study, a material-level simplification was carried out for changes in the material properties of the dam concrete due to calcium ion precipitation when the dam concrete was under the action of soft water erosion, without considering the evolution of the properties of the dam concrete under external loading and treating the concrete dam as a isotropic material. Based on the DuCOM calculation method, as shown in Figure 7, a simplified one-dimensional calcium ion transport model was developed to predict the durability of dam concrete in terms of calcium ion dissolution depth and strength change.

According to the DuCOM system and thematerial parameters from the design reports, the long-term properties of dam concrete were numerically analyzed. The mineral compositions of the cement, including C3A, C3S, C2S, C4AF, and Gypsum, were 15.50%, 54.76%, 14.16%, 5.47%, and 3.40% by mass, respectively. Because the mixture proportions of concrete varied during construction, average mixture proportions were applied for simulations to simplify the calculations, as shown in Table 3.

**Figure 8:**
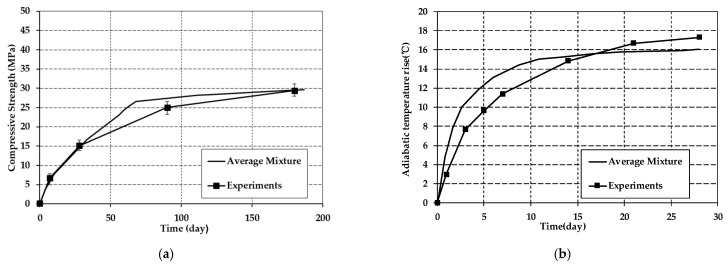
Comparison between experimental and simulated results: (**a**) Simulation results of compressive strength; (**b**) simulation results of adiabatic temperature rise.

The simulated compressive strength and adiabatic temperature rise were compared with the experiments, as shown in Figure 8.

The results indicate that the values of experiments are consistent with the simulated cases. Therefore, the application of average mix proportions to simulate the long-term properties of the concrete inside the dam is reasonable to some extend.

### 3.5. Numerical Analysis of Long-Term Properties of Dam Concrete

**Figure 9:**
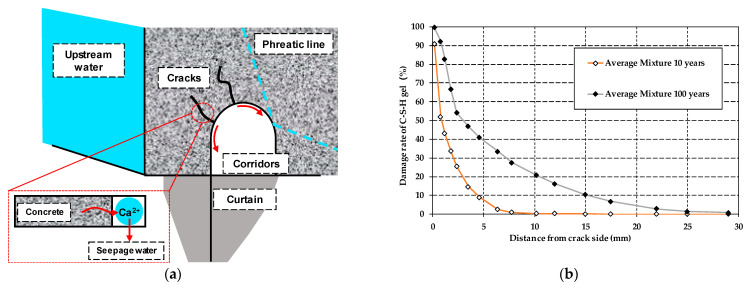
Long-term performance analysis of dam concrete: (**a**) the principle of calcium precipitation in the corridor; (**b**) long-term performance simulation of concrete dam.

The water seeping from the cracks takes more calcium ions away and accelerates the dissolution of concrete. This phenomenon will cause crack widths to increase and seriously endangers dam safety. Considering the above possibility, a one-dimensional FE model was set to simulate the process and was used to predict the long-term properties of dam concrete, as shown in Figure 9a. The boundary conditions were set as below. The crack side (at x = 0) was set to transfer heat, humidity, and calcium ions. The concentration was set to 1.455 mg/L, the same value as it was in the upstream reservoir. Time steps of 1 day were used in the first year and then increased to 10 days after that. The total calculation time was 36,500 days. The dissolution of C-S-H occurred after the loss of Ca(OH)_2_. The damage rate of C-S-H gel was used to represent the long-term durability of concrete, as shown in Figure 9b.

The contents of C-S-H gel in concrete have a strong relationship with strength and porosity. According to the simulated results, both the damage rate and depth are increased as time goes on. The calculation results show that after 10 years of corrosion reaction, the damage rate of C-S-H gel at 5 mm depth of the crack of the concrete surface is about 10%, and dam concrete has a certain degree of damage. After 100 years of dissolution reaction, the damage rate of C-S-H gel at 5 mm depth of the crack of the concrete surface is about 40%. The strength of concrete is significantly reduced. Moreover, as time goes on, the damage rate of C-S-H gel and the dissolution depth at cracks are both increasing. The calculation results show that the longer the dissolution time is, the more severe damage of C-S-H gel on the dissolving surface and internal concrete. In addition, the degree of soft water erosion is greater for the dam concrete. This article only provides a numerical analysis method for the prediction of the long-term durability of dam concrete. The specific warning value of the C-S-H gel damage rate is not in the scope of this article.

The numerical predictions indicate that after construction, in 10 to 100 years, the depth of dissolution damage increases from about 7 mm to 25 mm. According to the numerical calculation results, managers should complete reinforcement work, including grouting and sealing of cracks on time to ensure a dam’s long-term safety.

## 4. Conclusions

In this research, XPS, SEM, and multi-scale models of calcium leaching were used to evaluate the degree of damage to dam concrete due to calcium leaching. The following conclusions can be drawn from the present study:

(1) According to the water seepage and solid sediments in the corridor of a concrete dam, it is proved that there is a certain degree of calcium leaching in concrete dams according to a combination of various detection methods.

(2) A model of calcium leaching was developed, which considered the process of hydration and the development of pores. Compared with the experiments of former researchers’ studies, the model is verified. The results show that the model has high applicability for predicting the long-term durability of dam concrete.

(3) The numerical analyses using the calcium leaching model proposed in this paper can realize long-term durability prediction of dam concrete under erosion from soft water. The simulation shows that the depth of dissolution damage increases from about 7 mm to 25 mm after construction in 10 to 100 years.

(4) In this paper, the calculation results of the model can be combined with the damage rate of C-S-H gel to represent the long-term durability of concrete. In future studies, the changes in structural stress caused by corrosion reaction can be analyzed by combining structural calculations, and the threshold value of damage rate of C-S-H gel can be quantified to provide a basis for dam maintenance and reinforcement works.

## Figures and Tables

**Table 1:** Mixture proportions and mineral composition of cement.

Properties	Mixture Proportions (kg/m^3^)		Cement Composition (% Mass)				
	Water	Cement	C_3_A	C_3_S	C_2_S	C_4_AF	Gypsum
Values	653.3	1088.8	8.8	49.7	23.9	9.4	3.4

**Table 2:** Results of upstream and corridor water samples.

NO.	Sampling Location			pH	Average Value	Calcium Content (mg/L)	Average Value (mg/L)
	Section	Depth	Location				
1	#5	5 m	Dam surface near the upstream	6.72	6.58	1.450	1.455
2	#5	40 m		6.72		1.469	
3	#5	80 m		6.63		1.511	
4	#7	5 m		6.61		1.364	
5	#7	50 m		6.56		1.485	
6	#7	100 m		6.44		1.355	
7	#9	4 m		6.51		1.437	
8	#9	55 m		6.45		1.681	
9	#9	70 m		6.58		1.342	
10	#7	41.5 m	Corridor of dam foundation	10.43	10.42	22.100	21.370
11	#7	41.5 m		10.41		20.694	

**Table 3:** The average mixture proportions of dam concrete during construction.

Mixture Proportion (kg/m^3^)					
Water	Cement	Fly Ash	Slag	Fine Aggregate	Coarse Aggregate
85.2	75.1	167.6	5.6	732	1314

## Data Availability

The data presented in this study are available on request from corresponding author.
